# Supplementary material for: Targeting GOF p53 and c-MYC through LZK Inhibition or Degradation Suppresses Head and Neck Tumor Growth
Source: bioRxiv. 2024 Nov 20:2024.11.19.623840. Preprint. [Version 1] doi: 10.1101/2024.11.19.623840 (PMC11601640; doi:10.1101/2024.11.19.623840)
Supplement: 1 — Fig S1. LZK inhibitor diminishes growth of HNSCC cell lines. Fig S2. Kinase-dependent and -independent roles of LZK affect c-MYC and p53 abundance, respectively Fig S3. Inhibition of LZK impairs progression through the cell cycle Fig S4. Inhibition of LZK does not induce apoptosis in HNSCC cell lines Fig S5. GNE-3511 at 50 mg/kg is well tolerated in the PDX mouse models Fig S6. Compound #21 inhibits LZK activity and decreases viability of HNSCC cells Fig S7. Dependence of PROTAC-21A-mediated degradation of LZK involves both ubiquitin-like molecule NEDD8 and the proteasome Fig S8. TREEspot™ interaction maps for compound #21 and PROTAC-21A Fig S9. Shotgun proteomic analysis of total cell protein extracts from CAL33 TR LZK WT cell line following treatment with PROTAC97 Fig S10. LZK-targeting PROTAC reduces HNSCC viability but exhibits low membrane permeability Table S1. MAP3K13 amplification status for the HNSCC PDX models used in this study. Table S1. MAP3K13 amplification status for the HNSCC PDX models used in this study. Table S2. List of oligonucleotides used in this study. Table S3. Primer sets for RT-PCR analyses. Table S4. List of antibodies used for western blot analysis. Supplementary Figure S1. LZK inhibitor diminishes growth of HNSCC cell lines. A. RT-PCR analysis showing MAP3K13 transcripts of dox-inducible wild-type (LZK WT) or drug-resistant form (LZK Q240S) in stable CAL33 cell lines. B. Effect of GNE-3511 on colony formation of normal bronchial epithelial cells BEAS-2B. Data are representative of 3 independent experiments. C. Western blot analysis showing LZK and pJNK expression levels in dox-inducible wild-type (LZK WT) or drug-resistant LZK (LZK Q240S) expressing CAL33 cell lines. Data are representative of 3 independent experiments. D. Western blot showing the LZK dependence of the inhibitory effect of GNE-3511 on phosphorylation of JNK in 293T cells expressing either LZK WT or LZK Q240S. GAPDH served as the loading control. Data are representative [file NIHPP2024.11.19.623840v1-supplement-1.pdf]

bioRxiv preprint doi: https://doi.org/10.1101/2024.11.19.623840; this version posted November 20, 2024. The copyright holder for this preprint (which was not certified by peer review) is the author/funder. This article is a US Government work. It is not subject to copyright under 17 USC 105 and is also made available for use under a CC0 license.

Figure S1

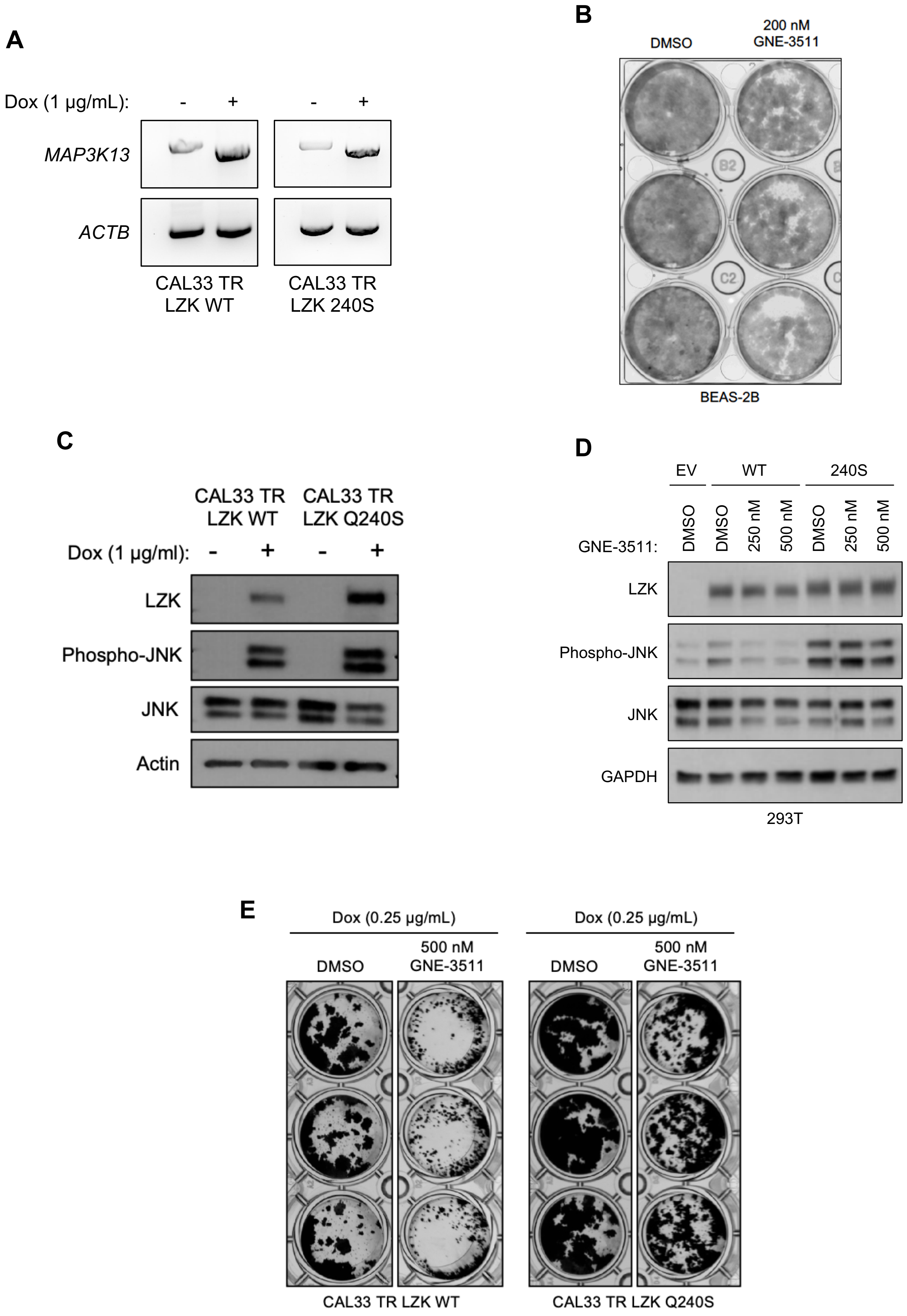

Supplementary Figure S1. LZK inhibitor diminishes growth of HNSCC cell lines. A. RT-PCR analysis showing *MAP3K13* transcripts of dox-inducible wild-type (LZK WT) or drug-resistant form (LZK Q240S) in stable CAL33 cell lines. **B.** Effect of GNE-3511 on colony formation of normal bronchial epithelial cells BEAS-2B. Data are representative of 3 independent experiments. **C.** Western blot analysis showing LZK and pJNK expression levels in dox-inducible wild-type (LZK WT) or drug-resistant LZK (LZK Q240S) expressing CAL33 cell lines. Data are representative of 3 independent experiments. **D.** Western blot showing the LZK dependence of the inhibitory effect of GNE-3511 on phosphorylation of JNK in 293T cells expressing either LZK WT or LZK Q240S. GAPDH served as the loading control. Data are representative of 3 independent experiments. EV, Empty Vector. **E.** Effect of GNE-3511 on colony formation of CAL33 cells with dox-induced LZK WT vs Q240S. Data are representative of 3 independent experiments.

bioRxiv preprint doi: https://doi.org/10.1101/2024.11.19.623840; this version posted November 20, 2024. The copyright holder for this preprint (which was not certified by peer review) is the author/funder. This article is a US Government work. It is not subject to copyright under 17 USC 105 and is also made available for use under a CC0 license.

Figure S2

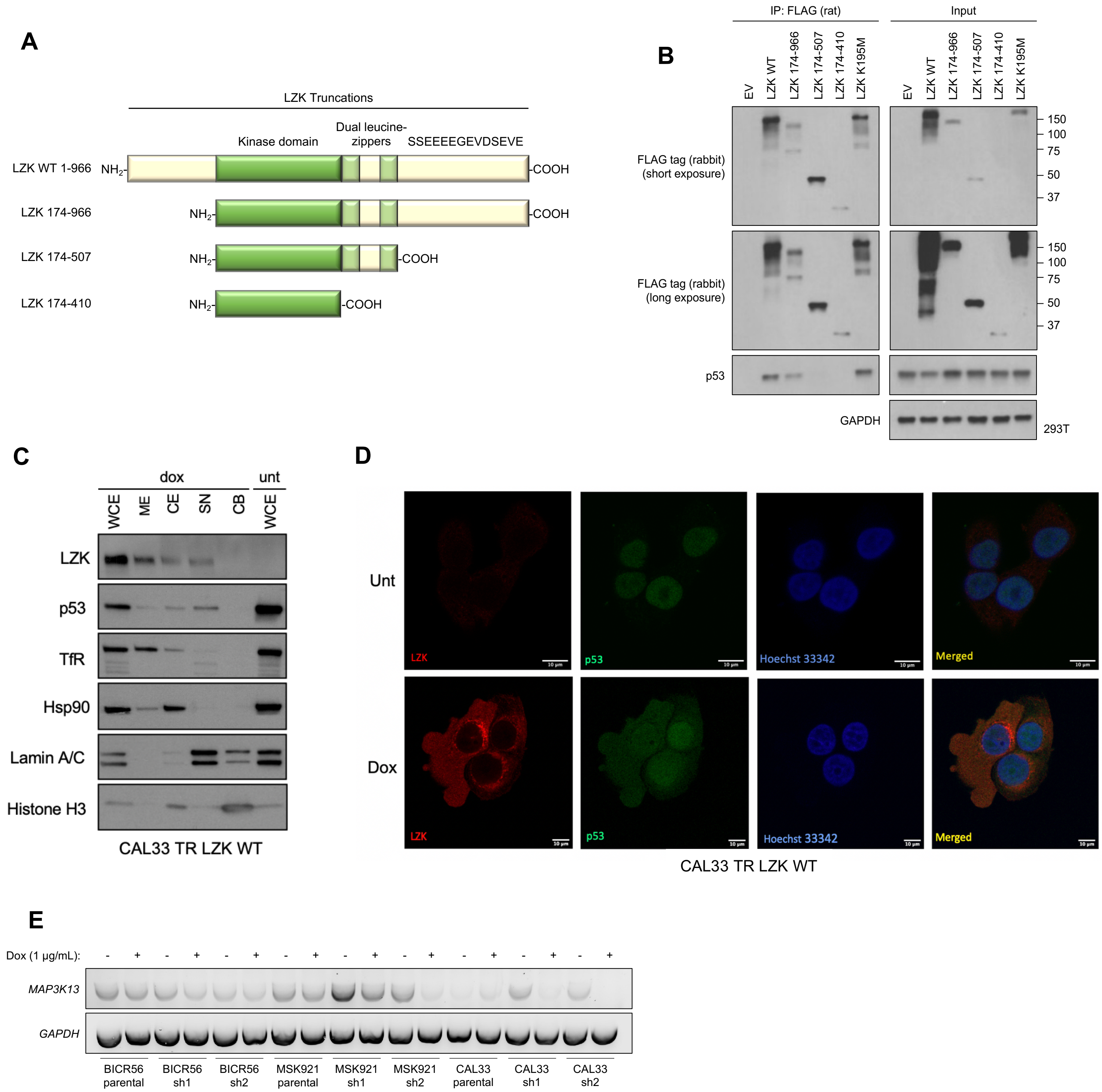

Supplementary Figure S2. Kinase-dependent and -independent roles of LZK affect c-MYC and p53 abundance, respectively. A. Schematic diagram of the N-terminal FLAG-tagged LZK and its truncation constructs tested for interaction with p53. **B.** Western blot showing interaction between p53 and the indicated FLAG-tagged LZK constructs as determined by co-immunoprecipitation with a FLAG antibody (left). Long and short exposures of the Western blot for FLAG-tagged LZK are shown. Input shows the abundance of the indicated proteins in the lysate used for co-immunoprecipitation. Data are representative of 3 independent experiments. **C.** Western blot showing LZK and GOF-p53 in membrane (ME), cytoplasmic (CE), soluble nuclear (SN) and chromatin-bound nuclear (CB) extracts from CAL33 TR LZK WT cells. Transferrin Receptor (TfR) served as the marker of ME compartment, Hsp90 as the marker of CE compartment, Lamin A/C as the marker of SN compartment, and Histone H3 as the marker of CB compartment. Data are representative of 3 independent experiments. **D.** Images of dox-induced CAL33 TR LZK WT cells stained for GOF-p53 (green) and LZK (red). The nuclei were stained with Hoechst 33342. Merged image shows colocalization. Scale bar = 10 μM. Data are representative of 3 independent experiments, each assessing 10 individual cells. **E.** RT-PCR analysis showing *MAP3K13* transcripts in the indicated parental cell lines or cell lines with dox-inducible shRNA targeting LZK. Data are representative of 3 independent experiments.

bioRxiv preprint doi: https://doi.org/10.1101/2024.11.19.623840; this version posted November 20, 2024. The copyright holder for this preprint (which was not certified by peer review) is the author/funder. This article is a US Government work. It is not subject to copyright under 17 USC 105 and is also made available for use under a CC0 license.

Figure S3

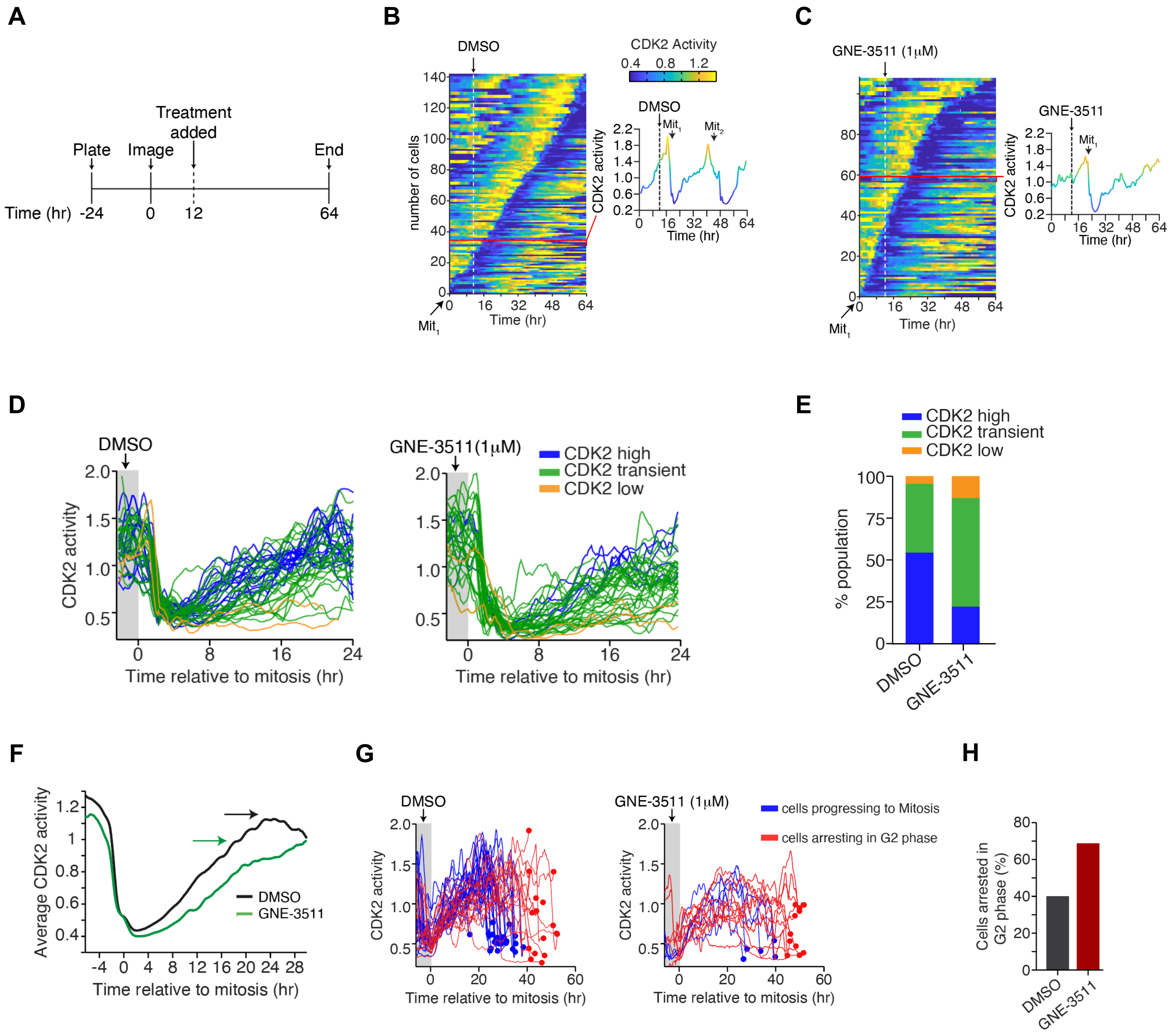

Supplementary Figure S3. Inhibition of LZK impairs progression through the cell cycle. A. Diagram of experimental protocol in SCC15 cells. Mitotic events were monitored using the peaks of CDK2 activity in each cell. Images were acquired 12 hours before exposure to treatment (DMSO or GNE-3511) and then every 12 minutes for 52 more hours. **B, C.** Heat maps of CDK2 activity in asynchronously cycling cells treated with DMSO (vehicle control) (B) or GNE-3511 (C) at the indicated time. Cells are sorted by the time of the first mitosis relative to the start of the imaging. Although the time of mitosis was independently identified using the nuclear marker H2B-mTurquoise, it can also be visualized by a rapid drop in CDK2 activity, as indicated by the black arrow. Inset is a CDK2 activity trace for a single representative cell. The red line indicates the position of that cell within the heat map. Mitotic events are noted as Mit_1 (first division after exposure), and Mit_2 (second division after exposure). Data are representative of 2 independent experiments. **D, E.** Effect of DMSO (vehicle control) and GNE-3511 on CDK2 activity throughout the cell cycle. Panel E represents a quantitative bar graph showing the percentage of the cell populations relative to CDK2 activity in treated cells. 2 independent experiments. **F.** Graph showing the effect of DMSO or GNE-3511 on average CDK2 activity during progression through the cell cycle. Arrows indicate maximum CDK2 activity reached. **G, H.** Graph showing the effect of DMSO and GNE-3511 on CDK2 activity in cells progressing to mitosis or arresting in G2-phase. Panel H represents a quantitative bar graph showing the percentage of cells arrested in G2 phase. Data are representative of 2 independent experiments.

bioRxiv preprint doi: https://doi.org/10.1101/2024.11.19.623840; this version posted November 20, 2024. The copyright holder for this preprint (which was not certified by peer review) is the author/funder. This article is a US Government work. It is not subject to copyright under 17 USC 105 and is also made available for use under a CC0 license.

Figure S4

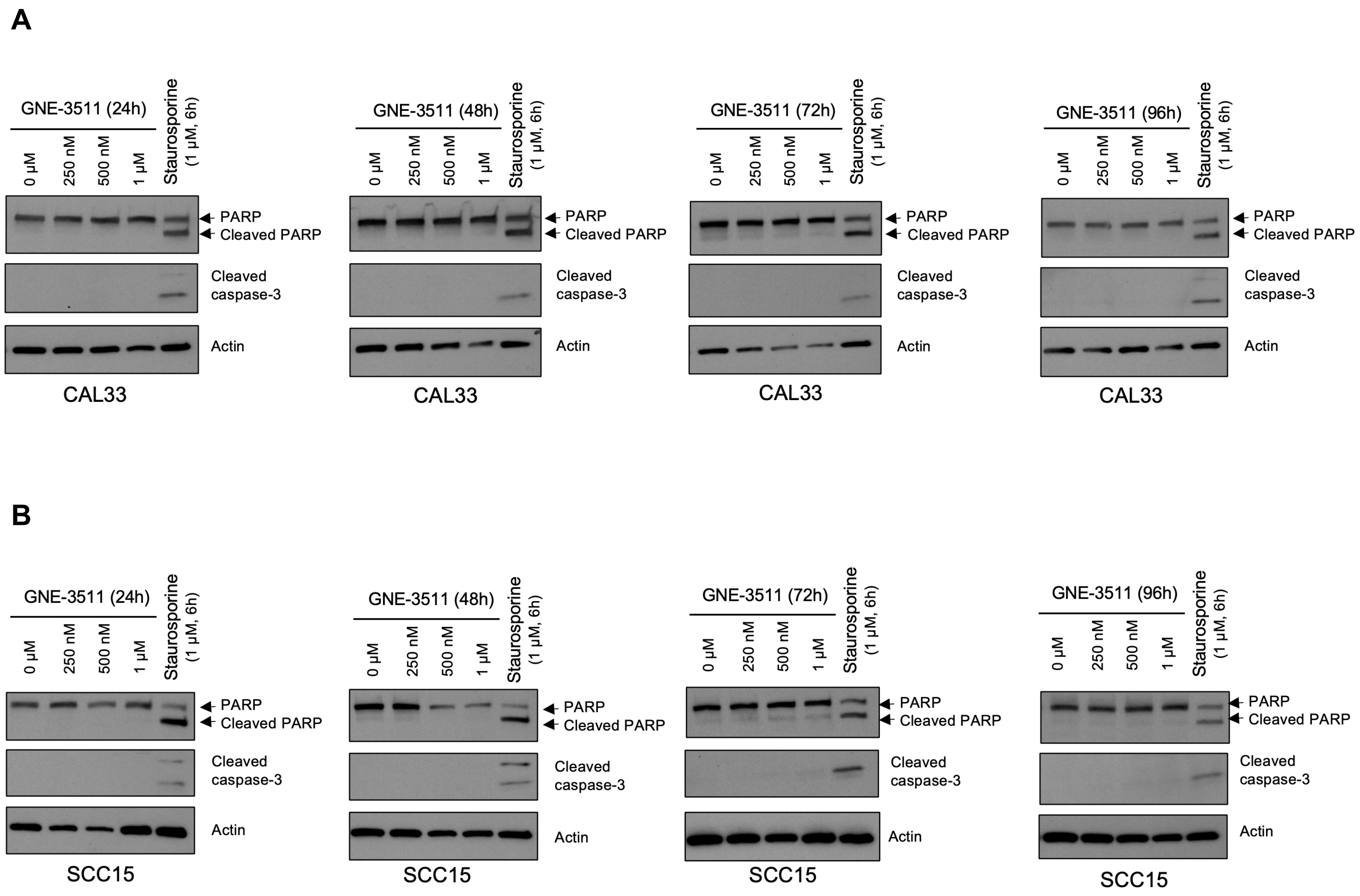

Supplementary Figure S4. Inhibition of LZK does not induce apoptosis in HNSCC cell lines. A, B. Western blots showing the effect of increasing concentrations of GNE-3511 treatment on apoptotic markers cleaved PARP and cleaved caspase-3 in CAL33 (A) and SCC15 (B) cells at different time-points (24h-96h). Six-hour treatment of 1 µM Staurosporine was used as a positive control to induce apoptosis. Actin served as a loading control. Data are representative of 3 independent experiments.

bioRxiv preprint doi: https://doi.org/10.1101/2024.11.19.623840; this version posted November 20, 2024. The copyright holder for this preprint (which was not certified by peer review) is the author/funder. This article is a US Government work. It is not subject to copyright under 17 USC 105 and is also made available for use under a CC0 license.

Figure S5

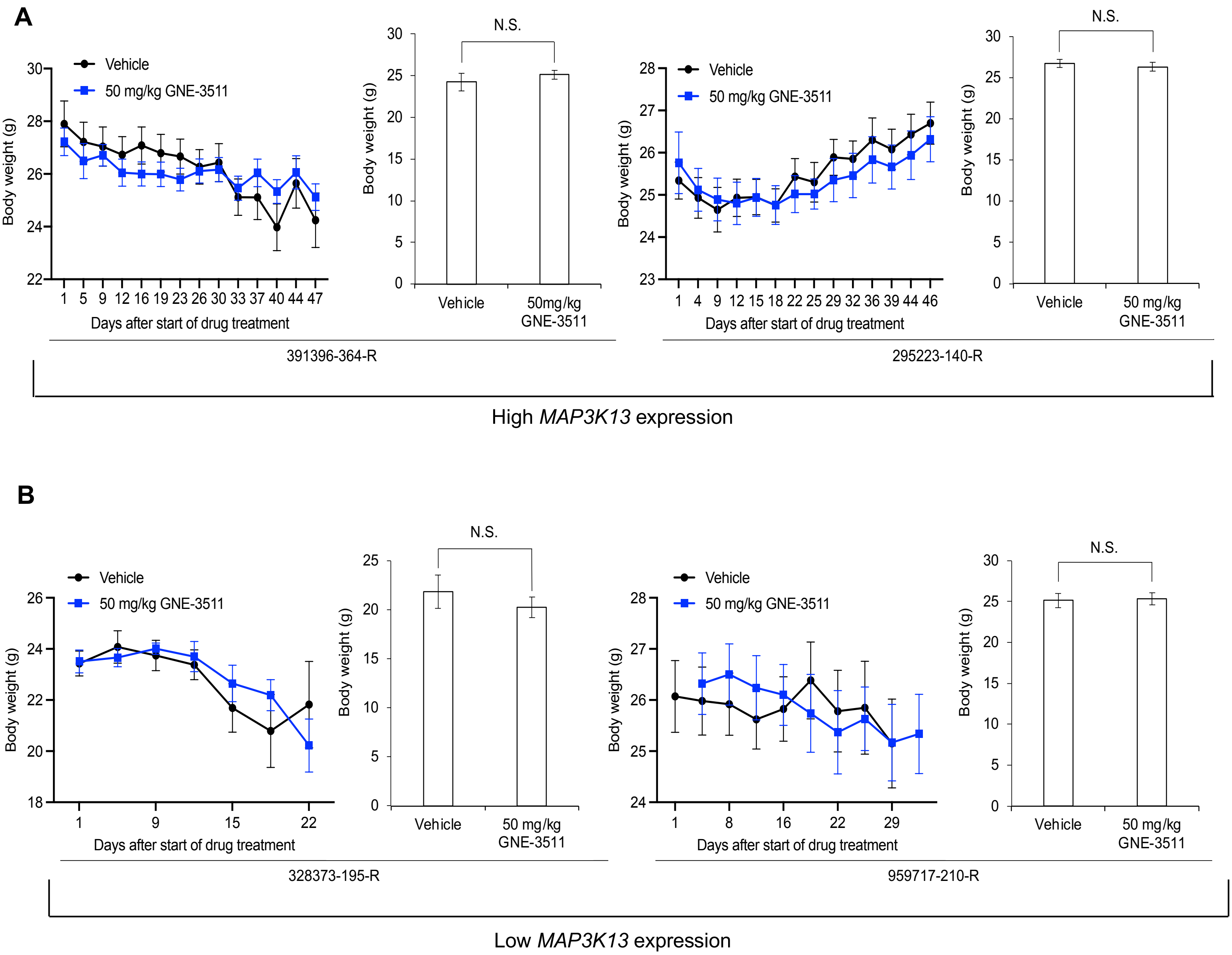

Supplementary Figure S5. GNE-3511 at 50 mg/kg is well tolerated in the PDX mouse models. A. Body weights of the mice for the HNSCC PDX mouse models harboring *MAP3K13* amplification (PDX #: 391396-364-R and 295223-140-R) treated with vehicle or GNE-3511 (50 mg/kg, q.d., five days on/two days off). Mean mouse body weight ± SEM are shown; Student's *t*-test; N.S., not significant. **B.** Body weights of the mice for the HNSCC PDX mouse models lacking amplification (PDX #: 328373-195-R and 959717-210-R) treated with vehicle or GNE-3511 (50 mg/kg, q.d., five days on/two days off). Mean mouse body weight ± SEM are shown; Student's *t*-test; N.S., not significant.

bioRxiv preprint doi: https://doi.org/10.1101/2024.11.19.623840; this version posted November 20, 2024. The copyright holder for this preprint (which was not certified by peer review) is the author/funder. This article is a US Government work. It is not subject to copyright under 17 USC 105 and is also made available for use under a CC0 license.

Figure S6

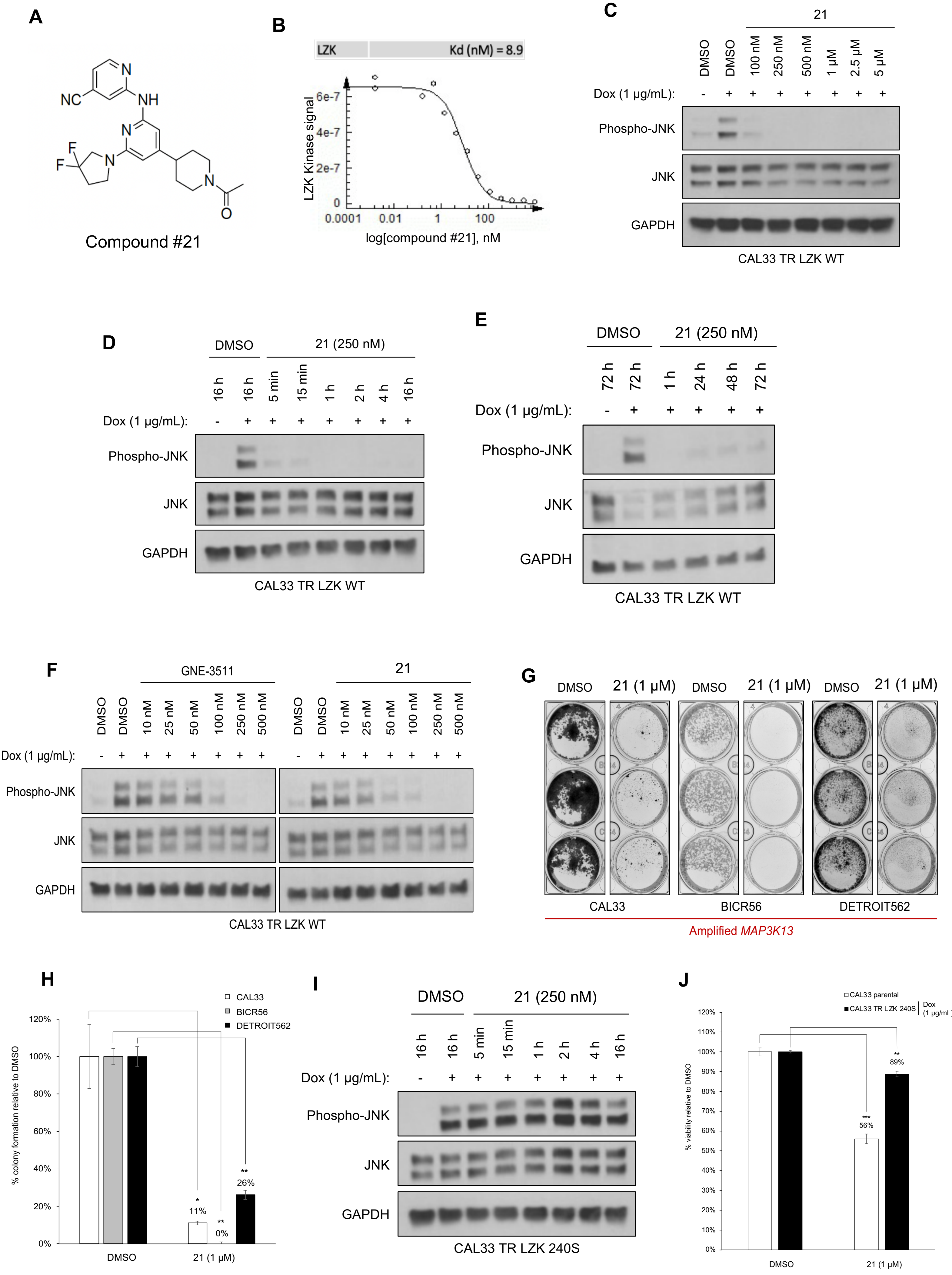

bioRxiv preprint doi: https://doi.org/10.1101/2024.11.19.623840; this version posted November 20, 2024. The copyright holder for this preprint (which was not certified by peer review) is the author/funder. This article is a US Government work. It is not subject to copyright under 17 USC 105 and is also made available for use under a CC0 license.

Supplementary Figure S6. Compound #21 inhibits LZK activity and decreases viability of HNSCC cells. A. Chemical structure of compound #21, the pharmacophore used in LZK-targeting PROTACs. **B.** Dose curve of the *in vitro* binding affinity of compound #21 to LZK assessed by Eurofin's KdELECT KINOMEscan^{TM} profiling. The amount of kinase measured by qPCR (Signal; y-axis) is plotted against the corresponding compound concentration in nM in log10 scale (x-axis). **C.** Western blot showing the effect of increasing concentrations of compound #21. LZK WT was induced with dox in CAL33 cells and the cells were exposed to the indicated concentration of #21 and phosphorylated JNK was monitored. GAPDH served as the loading control. Data are representative of 3 independent experiments. **D, E.** Western blots showing the effect of compound #21 over the indicated length of exposure in CAL33 LZK WT cells. Phosphorylated JNK was monitored. GAPDH served as the loading control. Data are representative of 3 independent experiments. **F.** Western blots showing the effect of GNE-3511 or compound #21 on phosphorylated JNK levels in dox-induced CAL33 LZK WT cells exposed to the indicated concentrations of drug. GAPDH served as the loading control. Data are representative of 3 independent experiments. **G, H.** Effect of compound #21 on colony formation of the indicated HNSCC cell lines. Panel G shows a representative experiment; panel H shows quantitative data for 3 experiments presented as the mean ± SEM; Student's *t*-test; $^{**}p < 0.01$, $^{*}p < 0.05$. **I.** Western blot showing the effect of compound #21 on phosphorylated JNK levels in CAL33 LZK^{Q240S} drug-resistant mutant cells exposed for up to 16 hours. GAPDH served as the loading control. Data are representative of 3 independent experiments. **J.** Effect of compound #21 on cellular viability in CAL33 cells with or without overexpression of LZK Q240S. Expression of LZK Q240S was induced with dox. Viability was assessed 72 hours after addition of compound #21 or DMSO (as the vehicle control) and determined by MTS assay. Data are shown as mean ± SEM for 3 experiments with triplicates each. Student's *t*-test; $^{***}p < 0.001$, $^{**}p < 0.01$.

bioRxiv preprint doi: https://doi.org/10.1101/2024.11.19.623840; this version posted November 20, 2024. The copyright holder for this preprint (which was not certified by peer review) is the author/funder. This article is a US Government work. It is not subject to copyright under 17 USC 105 and is also made available for use under a CC0 license.

Figure S7

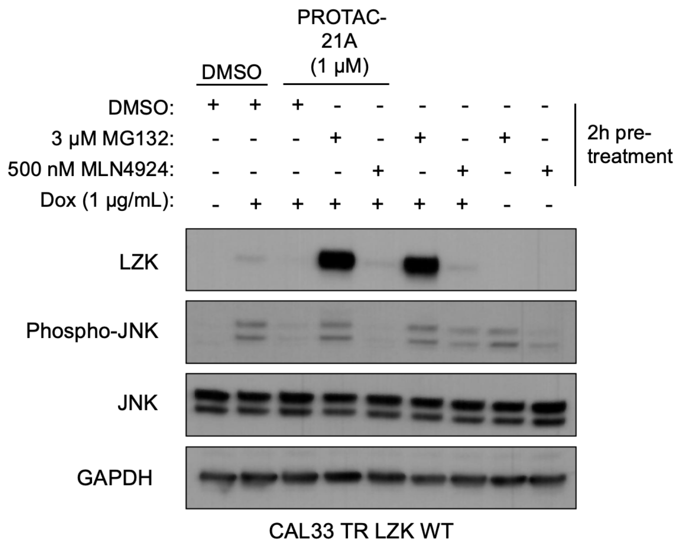

Supplementary Figure S7. Dependence of PROTAC-21A-mediated degradation of LZK involves both ubiquitin-like molecule NEDD8 and the proteasome. LZK WT was induced with dox in CAL33 cells and the cells were pre-treated for 2 hours with either MG132 or MLN4924 alone, or in combination with PROTAC-21A treatment for 24 h. Expression levels of LZK and phosphorylated JNK were monitored. GAPDH served as the loading control. Data are representative of 3 independent experiments.

bioRxiv preprint doi: https://doi.org/10.1101/2024.11.19.623840; this version posted November 20, 2024. The copyright holder for this preprint (which was not certified by peer review) is the author/funder. This article is a US Government work. It is not subject to copyright under 17 USC 105 and is also made available for use under a CC0 license.

Figure S8

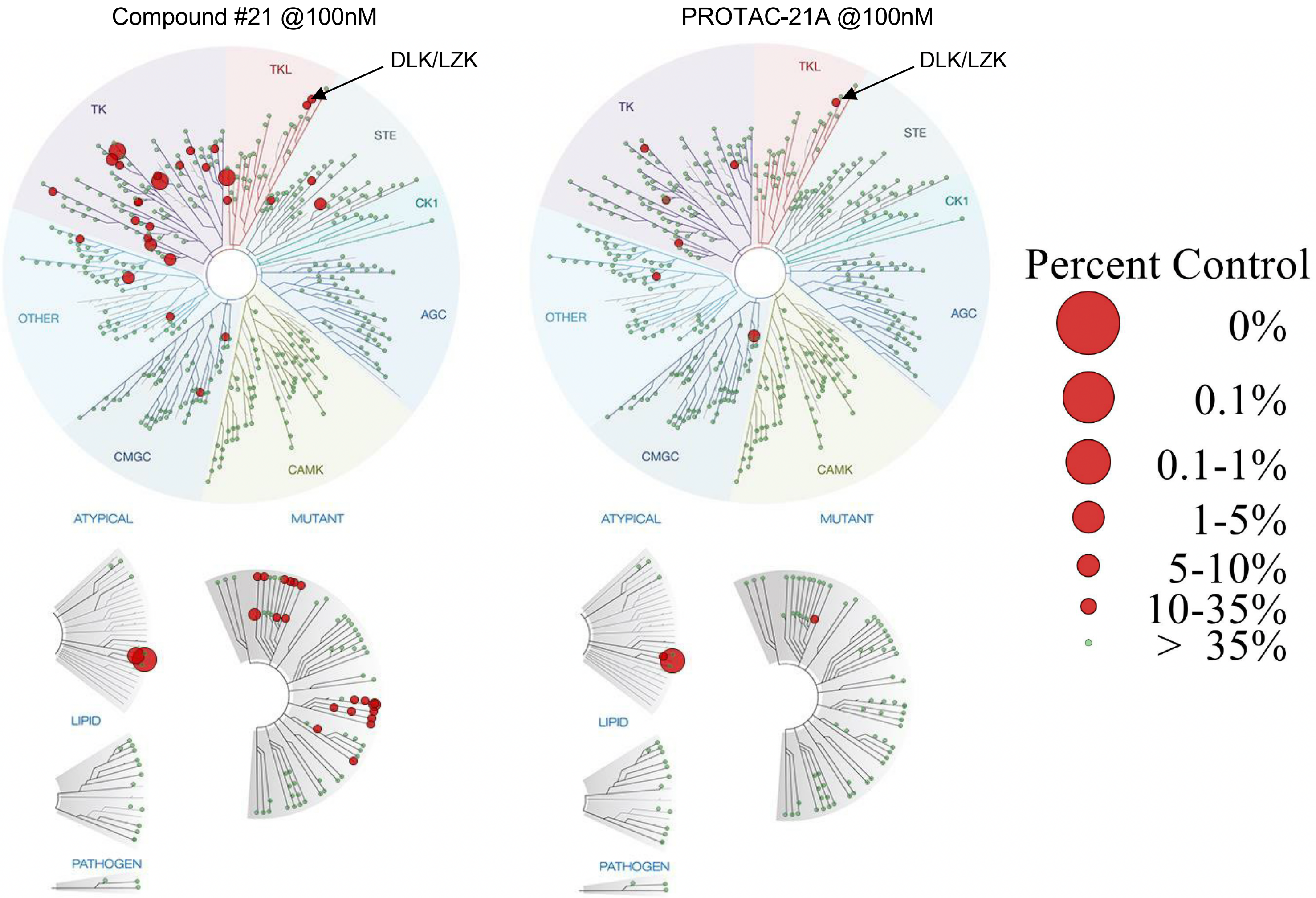

Supplementary Figure S8. TREE*spot*™ interaction maps for compound #21 and PROTAC-21A. KINOMEscan screening was performed for compound#21 and PROTAC-21A using an active site-directed ATP-independent competition binding assay by Eurofins discovery that quantitatively measures interactions between the two test compounds and more than 450 human kinases and disease relevant mutant variants. Both compounds were screened at 100nM, and results for primary screen binding interactions are reported as % Ctrl where lower numbers indicate stronger hits in the matrix. Kinases found to bind are marked with red circles, where larger circles indicate higher-affinity binding. Arrow indicates LZK/DLK binding. The kinomescan matrix for compound #21 and PROTAC-21A are represented in data file S1.

Figure S9

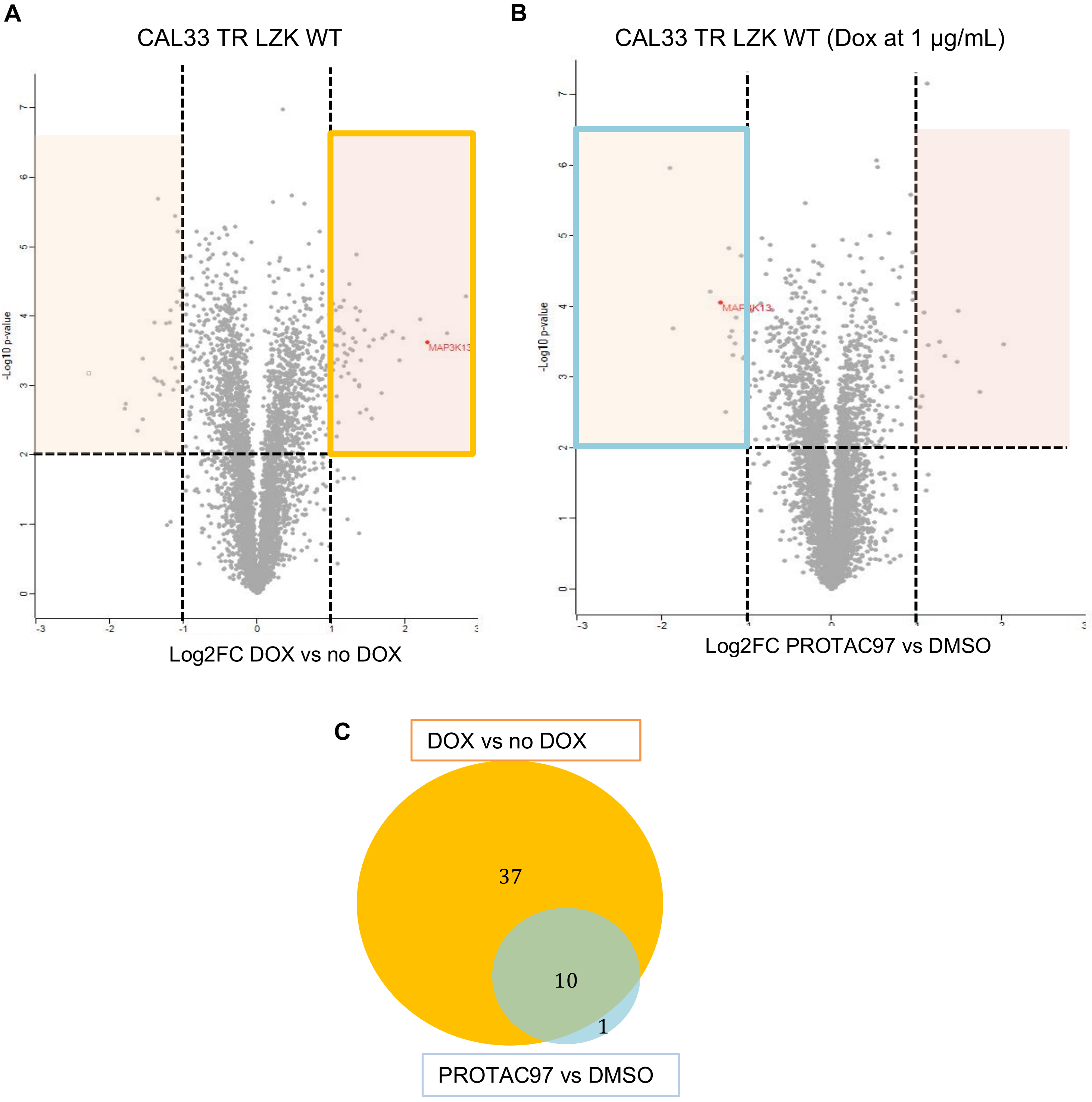

Supplementary Figure S9. Shotgun proteomic analysis of total cell protein extracts from CAL33 TR LZK WT cell line following treatment with PROTAC97. A, B. CAL33 TR LZK WT cells were treated with doxycyline (DOX) to induce LZK overexpression, followed by treatment with 1 µM PROTAC97 or DMSO control for 24 h. LZK protein is indicated in red. Volcano plot displaying the log2 fold change (Log2FC, x axis) against the t-test-derived −log10 statistical (n=3) p-value (y axis) for all proteins detected in the total cell extract from CAL33 TR LZK WT cells after DOX induction of LZK overexpression. The changes thresholds of Log2FC ≥ |1.0| and the significance threshold of –log10 p-value ≥ 2.0 were applied to identify proteins with levels decreased (green boxed area) or increased (red boxed area) in response to the DOX treatment (A). Volcano plot displaying the log2 fold change (Log2FC, x axis) against the t-test-derived −log10 statistical (n=3) p-value (y axis) for all proteins detected in the total cell extract from CAL33 TR LZK WT cells following induction of LZK overexpression and the treatment with PROTAC97 or DMSO. The changes thresholds of Log2FC ≥ |1.0| and the significance threshold of –log10 p-value ≥ 2.0 were applied to identify proteins with levels decreased (green boxed area) or increased (red boxed area) in response to the PROTAC97 treatment (B). **C.** Venn diagram illustrating the number of overlapping upregulated proteins from DOX vs. NO DOX (yellow boxed area on volcano plot A.) and downregulated proteins from PROTAC97 *vs*. DMSO (blue boxed area on volcano plot B.) MS analysis. The proteomic analysis of protein levels is represented in data file S2.

Figure S10

A

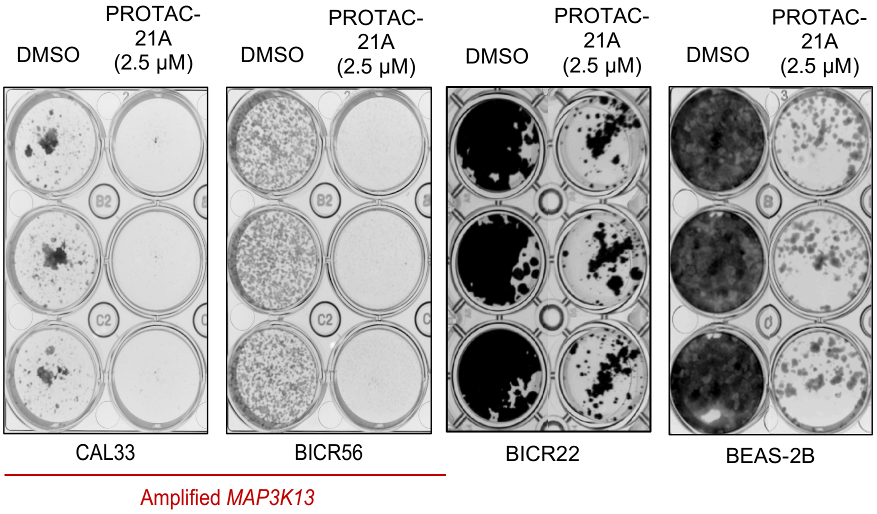

B

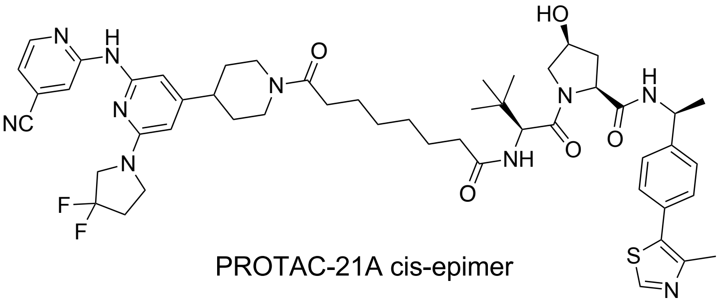

PROTAC-21A cis-epimer

C

PROTAC	Solubility (µg/mL)	PAMPA pH 7.4 (10^{-6} cm/sec)	RLM Stability (t1/2; min)
PROTAC-21A	<1	<1	>30
PROTAC-21C	26	<1	>30

D

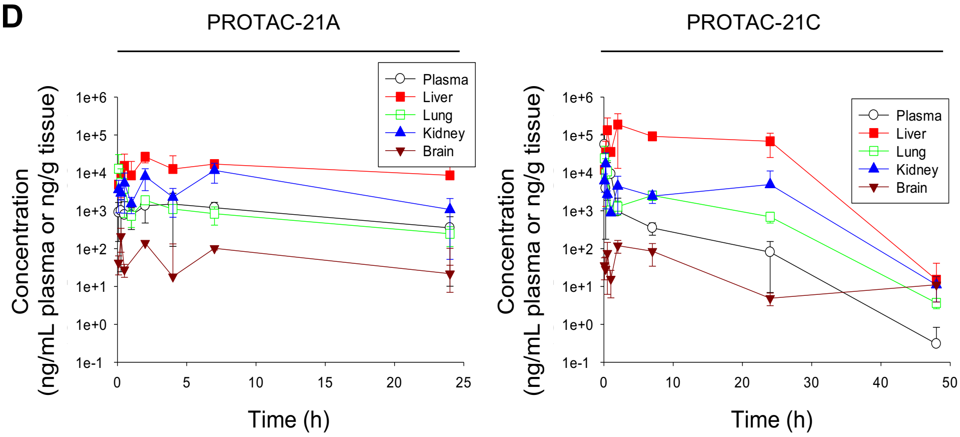

Figure S10. LZK-targeting PROTAC reduces HNSCC viability but exhibits low membrane permeability. A. Effect of PROTAC-21A on colony formation of the indicated cell lines. Data are representative of 3 independent experiments. **B.** Chemical structure of PROTAC-21A cis-epimer. **C.** *In vitro* ADME properties of PROTAC-21A and PROTAC-21C were assessed by kinetic solubility assay, parallel artificial membrane permeability assay (PAMPA) and rat liver microsomal (RLM) stability assay. **D.** Pharmacokinetic profiles of PROTAC-21A and PROTAC-21C were determined in female NSG mice after single IP administration of the PROTACs at 50 mg/kg. Mean concentration of each compound ± SD; n = 3 per time point.

Supplementary Table S1. *MAP3K13* amplification status for the HNSCC PDX models used in this study.

PDX model	Institute or Company	*MAP3K13* gene expression* (FPKM)	Disease classification
391396-364-R	NCI Patient-Derived Models Repository (PDMR)	12.94	Tongue (left base)
295223-140-R	NCI Patient-Derived Models Repository (PDMR)	12.43	Neck (left)
328373-195-R	NCI Patient-Derived Models Repository (PDMR)	2.62	Neck (left)
959717-210-R	NCI Patient-Derived Models Repository (PDMR)	1.6	Tongue (left)

*High *MAP3K13* expression is defined as FPKM>5
N/A, not applicable

Supplementary Table S2. List of oligonucleotides used in this study.

Primer	Sequence
LZK Q240S Forward (c718t_a719c_)	5'- CTGTGCCCATGGATCACTCTACGAGG -3'
LZK Q240S Reverse (c718t_a719c_)	5'- CCTCGTAGAGTGATCCATGGGCACAG -3'
LZK K195M Forward (a584t)	5'- GAGGTGGCCATCAAGAAAGTGAGAG -3'
LZK K195M Reverse (a584t)	5'- CTCTCACTTTCTTGATGGCCACCTC -3'
XbaI to start of LZK Forward	5'- TAATCTAGAATGGCCAACTTTCAGGAGCACCT -3'
NotI to end of LZK Reverse	5'- TTAGCGGCCGCTTACCAGGTAGCAGAGCTGTAGT -3'
T7 promoter	5'- TAATACGACTCACTATAGGG -3'
BGH reverse	5'- TAGAAGGCACAGTCGAGG -3'
XbaI to LZK kinase domain Forward	5'- TAATCTAGAATGCTGGGTAGTGGAGCCCAAGG -3'
NotI to LZK end kinase domain Reverse	5'- TTAGCGGCCGCTTAGGCAATGTCTAAATGCATGA -3'
NotI to LZK end zipper domains Reverse	5'- TTAGCGGCCGCTTACACTGCTTGCTCACGCTTAA -3'
NotI to LZK end stop codon Reverse	5'- TTAGCGGCCGCTTACCAGGTAGCAGAGCTGTAGT -3'

Supplementary Table S3. Primer sets for RT-PCR analyses.

Gene Symbol	Forward	Reverse
MAP3K13	AACTGATTCGAAGGCGCAGA	GGGCGTTTTCCAAGAGAGGA
ACTB	GGCACCACACCTTCTACAATG	GTGGTGGTGAAGCTGTAGCC
GAPDH	CCATGGAGAAGGCTGGGG	GTCCACCACCCTGTTGCTGTA

Supplementary Table S4. List of antibodies used for western blot analysis

Antibody	Source	Identifer
Rabbit anti-phospho-SAPK/JNK (Thr183/Tyr185) (81E11)	Cell Signaling Technology	Cat# 4668, RRID:AB_823588
Rabbit anti-SAPK/JNK	Cell Signaling Technology	Cat# 9252, RRID:AB_2250373
Rabbit anti-GAPDH (14C10)	Cell Signaling Technology	Cat# 2118, RRID:AB_561053
Rabbit anti-phospho-MKK7 (Ser271/Thr275)	Cell Signaling Technology	Cat# 4171, RRID:AB_2250408
Rabbit anti-MKK7	Cell Signaling Technology	Cat# 4172, RRID:AB_330914
Mouse anti-GST (26H1)	Cell Signaling Technology	Cat# 2624, RRID:AB_2189875
Rabbit anti-c-Myc (Y69)	Abcam	Cat# ab32072, RRID:AB_731658
Mouse anti-p53 (DO-1)	Santa Cruz Biotechnology	Cat# sc-126, RRID:AB_628082
Rabbit anti-LZK	YenZym Antibodies	Cat# YZ6696
Mouse anti-Transferrin receptor (H68.4)	Invitrogen	Cat#13_6800, RRID:AB_2533029
Rabbit anti-Hsp90 (C45G5)	Cell Signaling Technology	Cat#4877, RRID:AB_2233307
Mouse anti-Lamin A/C (4C11)	Cell Signaling Technology	Cat#4777, RRID:AB_10545756
Mouse anti-Histone H3 (96C10)	Cell Signaling Technology	Cat#3638, RRID:AB_1642229
Rabbit anti-PARP (46D11)	Cell Signaling Technology	Cat#9532, RRID:AB_659884
Rabbit anti-cleaved Caspase-3 (Asp175) (5A1E)	Cell Signaling Technology	Cat#9664, RRID:AB_2070042
Rat anti-FLAG (L5)	BioLegend	Cat# 637302, RRID:AB_1134268
Rabbit anti-FLAG (D6W5B)	Cell Signaling Technology	Cat# 14793, RRID:AB_2572291
Sheep anti-mouse IgG, secondary, HRP	GE Healthcare Life Sciences	Cat# NA931, RRID:AB_772210
Donkey anti-rabbit IgG, secondary, HRP	GE Healthcare Life Sciences	Cat# NA934, RRID:AB_772206
